# The Sense of Taste

**Published:** 1875-06

**Authors:** 


### The Sense of Taste.

The precise physiology of the sense of taste is a subject to which a good deal of attention has been devoted, but there still remains some obscure points to be solved in connection with it. It is well known that a great number of the sensations commonly included under the name of taste are in reality appreciated by the olfactory nerve. This is the case with nearly if not quite all of the aromatic and odorous flavors, as is clearly seen in cases of anosmia; whilst sour, salt, and astringent tastes are due to impressions on the nerves of common sensation, and are probably the results of chemical action. But the appreciation of "sweet" and "bitter" tastes is a special function, located in certain limited areas of the tongue, which are provided with special forms of papillæ, and to a small portion of the palate and fauces. Of these, the anterior portion of the tongue, close to the tip, and the posterior third of the dorsum are the most important, both possessing the power of discriminating sweet and bitter; but the anterior portion being apparantly more sensitive to sweet, the posterior to bitter tastes. The latter area is supplied by the glosso-pharyngeal nerve, which appears to act as the taste nerve; but with regard to the former, which is supplied by the lingual branch of the fifth, some doubt exists. Dr. A. Davidson, of Liverpool, has recently drawn attention to the subject in a paper, "On the Sense of Taste, and its Relation to Facial Paralysis and Anæsthesia," in which he discusses the various views which have been advanced, and relates three cases bearing upon the question. The results of clinical observation and of physiological experiment appear to show conclusively that the sense of taste in the anterior area is dependent upon the integrity of the chorda tympani, since injury to the fifth nerve, above the junction of the latter with it, although accompanied by complete anæsthesia of the side of the face and tongue, does not necessarily affect the sense of taste; whilst on the other hand, when the chorda tympani is injured or cut, taste is lost without loss of sensation. According to some, who regard the chorda tympani as a purely motor nerve, this is due to the affection of the functions of the sub-maxillary gland, or to the loss of power of erection of the taste papillæ. Neither of these explanations, however, appear to be satisfactory, and the weight of evidence would seem to be in favor of the view that some of the fibres of the nerve are afferent. In one of Dr. Davidson's cases there was complete paralysis of the portio dura on the left side, of deep origin, accompanied by entire loss of taste in the corresponding half of the tongue in the anterior part. In another case there was right facial paralysis from disease of the internal ear, and loss of taste on the right side of the tongue. In the third case there was complete anæsthesia of the parts supplied by the fifth nerve on the right side, probably resulting from a fracture of the skull; but although there was complete loss of sensation on that side of the tongue, the sense of taste was for some time unaffected. Its subsequent impairment is ascribed by Dr. Davidson to the changes in the mucous membrane of the tongue consequent upon impaired innervation. This case may serve to explain some anomalous cases in which injury to the fifth was associated with impairment of taste. Dr. Davidson believes that the taste nerves pass by the chorda tympani to the facial and thence probably by the nerve of Wrisberg to the brain.—*Lancet.*—*American Medical Weekly.*

